# Depression’s Shadow in First-Onset Schizophrenia: Mapping the Burden and Clinical Significance in Drug-Naïve Patients

**DOI:** 10.31083/AP53584

**Published:** 2026-10-26

**Authors:** Lin Zhang, Jie Zhang, Lu Wen, Xinming Pan, Hongjun Mao, Xiao-E Lang, Junjun Liu, Xiangyang Zhang

**Affiliations:** ^1^Department of Psychiatry, The Second People’s Hospital of Jiangning District, 211103 Nanjing, Jiangsu, China; ^2^Department of Psychiatry, First Hospital of Shanxi Medical University, 030001 Taiyuan, Shanxi, China; ^3^Department of Psychiatry, Nanjing Meishan Hospital, 210039 Nanjing, Jiangsu, China; ^4^CAS Key Laboratory of Mental Health, Institute of Psychology, Chinese Academy of Sciences, 100000 Beijing, China

**Keywords:** schizophrenia, severe depressive symptom, comorbidity, psychotic symptom, suicide attempt

## Abstract

**Background:**

Severe depressive symptoms (SDS) are common in patients with schizophrenia (SZ) and are associated with poor prognosis, reduced quality of life, and an increased risk of suicide. However, the prevalence of SDS and its key associated factors in first-onset drug-naïve patients with SZ remain unclear.

**Methods:**

This cross-sectional study enrolled 320 inpatients diagnosed with SZ according to the Diagnostic and Statistical Manual of Mental Disorders, Fifth Edition (DSM-5), including 164 males and 156 females, aged 18 to 60 years, with a mean disease duration of 2.18 years. The severity of psychotic symptoms was assessed using the Positive and Negative Syndrome Scale (PANSS), anxiety symptoms were evaluated using the Hamilton Anxiety Rating Scale (HAMA), and SDS were assessed using the Hamilton Depression Rating Scale (HAMD). A history of suicide attempts was obtained through standardized interviews. The Kolmogorov-Smirnov test was used to assess the normality of continuous variables. Bonferroni correction was applied to all between-group comparisons of continuous and categorical variables. Univariate analyses and multivariable logistic regression were performed to identify factors independently associated with SDS. A sensitivity analysis excluding patients with a disease duration greater than 12 months was conducted to assess the robustness of the findings.

**Results:**

Among the 320 patients with SZ, 159 (49.7%) met the criteria for comorbid SDS. After adjustment for age, sex, disease duration, and marital status, multivariable logistic regression analysis demonstrated that higher PANSS scores (OR = 1.045, 95% CI: 1.024 to 1.067, *p* < 0.001), higher HAMA scores (OR = 1.284, 95% CI: 1.198 to 1.376, *p* < 0.001), and a history of suicide attempts (OR = 1.513, 95% CI: 1.222 to 1.875, *p* < 0.001) were independently associated with comorbid SDS. Excluding patients with a disease duration greater than 12 months (n = 138) did not materially alter the results. Furthermore, a combined model incorporating these three variables demonstrated excellent discriminatory performance, with an area under the receiver operating characteristic curve (AUC) of 0.937 (95% CI: 0.904 to 0.958, *p* < 0.001).

**Conclusions:**

Severe depressive symptoms are highly prevalent among first-onset, drug-naïve patients with schizophrenia and are independently associated with more severe psychotic symptoms, greater anxiety severity, and a history of suicide attempts. These findings provide important evidence to support early screening and targeted interventions for this vulnerable patient population.

## Main Points

1. This cross-sectional study investigated the prevalence and independent influencing factors of severe depressive symptoms among first-episode, drug-naïve patients with schizophrenia, filling the research gap in this specific population.

2. This study confirmed that severe psychotic symptoms, anxiety symptoms, and a history of suicide attempts were independently positively associated with comorbid severe depressive symptoms in schizophrenia patients.

3. The combined model incorporating Positive and Negative Syndrome Scale (PANSS) score, Hamilton Anxiety Rating Scale (HAMA) score and suicide attempt history achieved excellent discriminatory ability for severe depressive symptoms, which supports early screening and targeted intervention in clinical practice.

## 1. Introduction

Schizophrenia (SZ) is a group of chronic and severe mental disorders characterized by disorders of perception, thought, emotional and behavior, accompanied by significant impairment of social functions, that affects approximately 0.6% of the global population [1]. This chronic condition imposes a substantial global disease burden, causing severe impairments in patients’ social, occupational, and daily living functions, which leads to long-term disability, and the generation of considerable psychological distress for both patients and their families [2]. Notably, depressive symptoms, traditionally recognized as core features of mood disorders, represent a highly prevalent yet frequently overlooked comorbidity in SZ and are often overshadowed by more prominent psychotic symptoms such as hallucinations and delusions [3,4,5]. Epidemiological studies have consistently demonstrated that depressive symptoms are common in SZ patients, with reported prevalence ranging from 30–50% during the disease course [6], and up to 80% of patients may experience at least one clinically significant depressive episode [7]. Severe depressive symptoms (SDS), in particular, are closely associated with poorer clinical outcomes, including higher rates of suicide attempts and suicide-related behaviors (20–50% of SZ patients report suicide attempts during their illness) [8], reduced quality of life [9], further deterioration of social functioning, increased risk of relapse, and unfavorable long-term prognosis [3].

Despite the high prevalence and serious clinical consequences of comorbid depressive symptoms in SZ, the true prevalence of comorbid SDS remains unclear. This is particularly true in first-episode drug-naïve (FEDN) SZ patients, a specific population with no history of antipsychotic medication use. This absence of prior treatment effectively eliminates potential confounding effects of antipsychotic drugs on depressive symptoms, including drug-induced depression and side effects (such as emotional blunting) that may mask depressive symptoms. Compared with chronic SZ patients, FEDN individuals more accurately reflect the intrinsic depressive characteristics of SZ itself, avoiding the confounding that can occur between disease-related depression and drug-related mood changes. Thus, studying this population provides a reliable foundation for exploring the intrinsic association of comorbid SDS in SZ. Although previous studies have extensively examined depressive symptoms in SZ, several limitations are evident. First, most existing studies primarily involved patients with chronic SZ or those receiving long-term antipsychotic treatment, making it difficult to disentangle the independent effects of the illness itself from medication effects on depressive symptoms. Second, previous studies lacked consistency in defining depressive symptoms and the assessment tools employed. Many studies focused only on general depressive symptoms without specifically emphasizing SDS, resulting in inconsistent and incomparable findings. Third, most previous studies had limited sample sizes or high heterogeneity among study populations, reducing the representativeness and reliability of the results. Consequently, prevalence data on comorbid SDS in FEDN SZ patients remain insufficient.

Beyond the uncertainty regarding prevalence, accumulating evidence has identified multiple potential correlates of depressive symptoms in patients with SZ, encompassing demographic, clinical, biological, and psychosocial domains. From a demographic perspective, factors such as female sex [10], younger age at onset [11], lower educational attainment [12], and unmarried or divorced status [10] have been identified as potential risk factors. Clinically, the severity of mental symptoms, whether positive (such as hallucinations and delusions) or negative (such as anhedonia and social withdrawal), is closely related to depressive symptoms. More severe mental symptoms are associated with a higher incidence of comorbidities and depression [13,14].

However, the majority of those studies were conducted in patients with chronic SZ or who had received pharmacological treatment, and evidence regarding such associations in patients with FEDN remains limited and inconsistent. Research findings are contradictory regarding the direction and magnitude of those associations, particularly for psychotic symptoms, cognitive function, and inflammatory markers. Given this gap, the present study aimed to address two core objectives: (1) determine the prevalence of comorbid SDS in patients with FEDN SZ and (2) to explore potential risk factors associated with SDS comorbidity in this specific population. The findings are expected to provide evidence-based support for the early identification, clinical intervention, and individualized treatment of SDS in drug-naïve patients with SZ.

## 2. Materials and Methods

### 2.1 Participants

This study (ID: H18031) was approved by the Ethics Committee of the Institute of Psychology, Chinese Academy of Sciences. A total of 320 patients (164 males and 156 females, mean age of 26.96 ± 9.76 years, average disease duration of 2.18 years) with FEDN SZ were included in the analysis.

**Inclusion criteria:** (1) Age between 18 to 60 years; (2) First episode of illness with no prior antipsychotic treatment; and (3) Diagnosis of SZ according to the Diagnostic and Statistical Manual of Mental Disorders, Fifth Edition (DSM-5) criteria, confirmed by the Structured Clinical Interview for DSM-5 (SCID-5).

**Exclusion criteria: **(1) Comorbid organic brain disease (e.g., organic brain lesions, epilepsy); (2) Substance dependence or abuse; (3) Severe personality disorder; (4) Schizoaffective disorder; and (5) Pregnancy or lactation. All participants were fully informed of the research process before enrollment and provided written informed consent.

### 2.2 Data Collection and Scale Evaluation

Demographic and clinical information was recorded for each participant, including sex, age, duration of illness, educational level, marital status, history of suicidal behavior, and scores on the Hamilton Depression Rating Scale (HAMD), Hamilton Anxiety Rating Scale (HAMA), and Positive and Negative Syndrome Scale (PANSS), as well as fasting blood glucose and lipid levels.

In the early morning, before food, relevant blood samples were collected, processed, and analyzed in the hospital laboratory prior to 11 a.m the same day. Plasma glucose, triglyceride, low and high-density lipoprotein cholesterol, and total cholesterol levels were assessed using an ARCHITECT c8000 biochemical analyzer (Abbott Laboratories, Abbott Park, Illinois, USA).

Depressive symptoms were assessed using the 24-item HAMD. Depression ranging from 8–19 is classified as mild depression, 20–34 as moderate depression, and over 35 as severe depression. A total score of >35 was used as the SDS cutoff [15].

Anxiety symptoms were evaluated with the HAMA, which assesses both somatic and psychic anxiety. Scores of 18–24 indicate mild-to-moderate anxiety, while scores of 25–30 indicate moderate-to-severe anxiety (maximum score: 56) [16].

Psychotic symptoms were assessed using the PANSS, which comprised three subscales: Positive, negative, and general psychopathological. The positive subscale evaluated psychopathological symptoms such as delusions and hallucinations; the negative subscale measured deficit symptoms, including emotional blunting and social withdrawal; and the general psychopathology subscale covered associated symptoms such as anxiety and disorientation. Higher scores indicate more severe symptoms [17].

Clinical assessments were conducted by two experienced psychiatrists. Inter-rater reliability was high for the total HAMD and HAMA scores, with correlation coefficients of 0.85 and 0.84, respectively. For the PANSS total score, inter-rater reliability was also assessed, yielding an intraclass correlation coefficient (ICC) of 0.82.

Suicide attempt (SA) was defined as self-harm or self-injurious behavior in which the patient had at least some intention of ending their life [18]. Assessment was conducted via clinical interview, beginning with the question, “Have you ever, at any time in your life, attempted suicide?” An affirmative response was followed by further inquiries regarding the frequency, method, and timing. When clarification was needed, information from family members, relatives, or close friends was used to supplement participant responses.

### 2.3 Statistical Analysis

SPSS version 26.0 (IBM Corporation, Armonk, NY, USA) was utilized for all statistical analyses. The Kolmogorov-Smirnov test was used to evaluate the normality of continuous variables. Categorical and continuous variables were compared between groups with chi-square and two-sample *t*-tests, respectively. All between-group comparisons (continuous and categorical variables) were corrected by the Bonferroni method. The adjusted alpha level was 0.05/12 ≈ 0.004167. Linear regression assessed collinearity, utilizing a variance inflation factor (VIF) threshold of five. Multiple logistic regression identified independent influencing factors associated with comorbid depression in SZ. The Area Under the Curve (AUC) of the receiver operating characteristic curve was applied to assess the diagnostic performance of significant variables in distinguishing SZ patients complicated with SDS, with values of 0.7–0.8 indicating acceptable model performance. Statistical significance was set at *p* < 0.05 (two-tailed). Sensitivity analysis excluded patients with a disease course of more than 12 months to test robustness. Using the continuous 24-item Hamilton Depression Rating Scale (HAMD-24) total score in all regression models.

The sample size was calculated using G*Power 3.1.9.2 software (http://www.softpedia.com/get/Science-CAD/G-Power.shtml) [19]. Based on previous epidemiological evidence indicating that the prevalence of comorbid depressive symptoms in SZ ranges from 30–50% [6], a reference prevalence of 40% was employed. With power = 0.80 and *α* = 0.05, the sample size calculation suggested that at least 220 patients were needed to achieve sufficient statistical power.

## 3. Results

### 3.1 Comparison of Clinical and Demographic Characteristics

A total of 320 patients with SZ were enrolled in the present study. The prevalence of SDS in this population was 49.7% (*n* = 159/320).

Significant between-group differences were detected in HAMA and PANSS scores, Positive scale score, Negative scale score, General psychopathological scale score, smoking status, and suicide attempt history (all *p* < 0.001). Furthermore, patients in the SDS group exhibited significantly elevated of low-density lipoprotein cholesterol (LDL-C, *p* = 0.005) and fasting blood glucose (FBG, *p* = 0.014). No significant differences were observed in age, gender, disease duration, marital status, body mass index (BMI), or high-density lipoprotein cholesterol (HDL-C) levels between the two groups (Table 1).

**Table 1.** **The sociodemographic and clinical characteristics of SZ patients with or without SDS**.

Variable		SDS group (n = 159)	NSDS group (n = 161)	*t/*χ^2^/*U*	*p*
Age		27.06 ± 10.31	26.86 ± 9.21	0.183	0.855
Duration of illness (years)		2.35 ± 1.77	2.01 ± 1.59	1.791	0.074
HAMA		34.84 ± 5.62	24.97 ± 5.66	15.661	<0.001^*^
PANSS		153.94 ± 16.85	130.25 ± 18.53	11.957	<0.001^*^
Positive scale score		29.46 ± 5.15	25.59 ± 6.55	5.868	<0.001^*^
Negative scale score		36.3 ± 5.19	30.26 ± 7.23	8.573	<0.001^*^
General psychopathological scale score		73.33 ± 9.8	61.75 ± 9.63	10.664	<0.001^*^
FBG (mmol/L)		5.36 ± 0.79	5.13 ± 0.86	2.474	0.014
HDL-C (mmol/L)		1.28 ± 0.32	1.23 ± 0.34	1.289	0.198
LDL-C (mmol/L)		2.74 ± 0.83	2.50 ± 0.72	2.829	0.005
BMI		23.94 ± 3.45	23.18 ± 3.84	1.845	0.066
Gender				0.617	0.44
	Male	85 (53.5%)	79 (49.1%)		
	Female	74 (46.5%)	82 (50.9%)		
Marital status				0.132	0.717
	Unmarried	96 (60.4%)	94 (58.4%)		
	Married	63 (39.6%)	67 (41.6%)		
Smoking history				11.894	0.001^*^
	Yes	52 (32.7%)	26 (16.1%)		
	No	107 (67.3%)	135 (83.9%)		
Suicidal attempt				58.445	<0.001^*^
	Yes	106 (66.7%)	40 (24.8%)		
	No	53 (33.3%)	121 (75.2%)		

Note: HDL-C, High-density lipoprotein cholesterol; BMI, Body mass index; SZ, Schizophrenia; SDS, Severe depressive symptoms; NSDS, Non-severe depressive symptoms; PANSS, Positive and Negative Syndrome Scale; HAMA, Hamilton anxiety rating scale; FBG, Fasting blood glucose; LDL-C, Low density lipoprotein cholesterol. The symbol "*" indicates statistical significance after Bonferroni correction. Given 12 independent between group comparisons, the adjusted threshold was computed as 0.05 divided by 12, corresponding to *p* < 0.004167.

### 3.2 Analysis of Factors Associated With Severe Depressive Symptoms in SZ Patients

Table 2 illustrates the relationships between SDS and demographic, clinical, and biochemical indicators in patients with SZ. Multivariate logistic regression analysis was conducted to explore potential risk factors for comorbid SDS, using SDS status as the dependent variable and variables with *p* < 0.05 in univariate analysis as independent factors.

**Table 2.** **Multivariate logistic regression analysis of demographic and clinical related factors in SZ patients with or without SDS**.

Variable	B	SE	Wald	OR	95% CI	*p*
Age	0.009	0.021	0.195	1.009	0.969–1.052	0.659
FBG	0.088	0.208	0.177	1.092	0.726–1.643	0.674
LDL-C	0.084	0.251	0.112	1.088	0.665–1.779	0.738
PANSS	0.044	0.01	17.969	1.045	1.024–1.067	<0.001
Positive scale score	0.009	0.064	0.022	1.009	0.891–1.144	0.883
Negative scale score	0.05	0.025	4.21	1.052	1.002–1.103	0.04
General psychopathological scale score	0.022	0.063	0.117	1.022	0.903–1.157	0.732
HAMA	0.250	0.035	49.854	1.284	1.198–1.376	<0.001
Suicidal attempt	0.414	0.109	14.371	1.513	1.222–1.875	<0.001
Smoking history	0.723	0.437	2.73	2.06	0.874–4.854	0.098

Note: HAMA, Hamilton anxiety rating scale; FBG, Fasting blood glucose; LDL-C, Low density lipoprotein cholesterol; B, Unstandardized regression coefficient; SE, Standard error of the regression coefficient; OR, Odds ratio.

The results indicated that HAMA scores (OR = 1.284, 95% CI: 1.198–1.376, *p* < 0.001), PANSS total scores (OR = 1.045, 95% CI: 1.024–1.067, *p* < 0.001), Negative scale score (OR = 1.052, 95% CI: 1.002–1.103, *p* = 0.04), and suicide attempts (OR = 1.513, 95% CI: 1.222–1.875, *p* < 0.001) were significantly and positively associated with SDS (Table 2). Excluding patients with a disease course of more than 12 months (*n* = 138) did not substantially change the results. Linear regression analysis showed that the total score of HAMD was significantly correlated with the negative subscale of PANSS (β = 0.167, *p* < 0.001), HAMA total score (β = 0.847, *p* < 0.001), and suicide risk (for every 1-point increase in OR = 1.038, *p* < 0.001).

### 3.3 Independent Factors Associated With Comorbid Severe Depressive Symptoms in SZ: ROC Curve Analysis

The receiver operating characteristic (ROC) curve analysis was performed to evaluate the association between influencing factors and the occurrence of comorbid SDS in SZ (Fig. 1). SA, Negative scale score (N score), PANSS total score, and HAMA score were significantly associated with SDS occurrence, with corresponding AUC values of 0.719, 0.756, 0.827, and 0.890, respectively. The combined model incorporating these three indicators achieved an AUC of 0.937 for discriminating patients with comorbid severe depressive symptoms (Fig. 1). The optimal threshold values were determined according to the maximum Youden index. For the total PANSS score, the optimal cutoff value was 135.50, yielding a sensitivity and specificity of 87.4% and 51.1%, respectively. For Negative scale score (N score), the optimal cutoff was identified as 33.5, with 73% sensitivity and 41% specificity. For HAMA scores, the optimal cutoff was identified as 30.50, with 81.8% sensitivity and 65.6% specificity. Regarding suicide attempt history, the corresponding optimal cutoff was 1.50, with a sensitivity of 66.7% and a specificity of 42.3%.

**Fig. 1.**
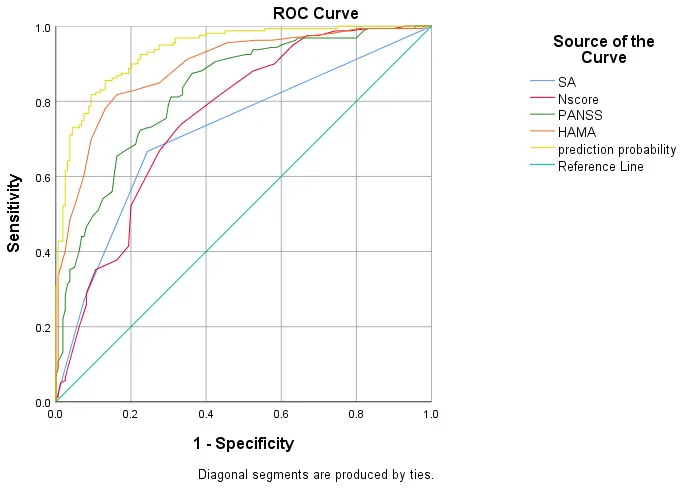
**Suicide attempt (SA), Negative scale score (N score), PANSS total score, and HAMA scorewere significantly associated with comorbid SDS occurence, with corresponding AUC values of 0.719, 0.756, 0.827, and 0.890, respectively**. The combined model incorporating these three indicators achieved an AUC of 0.937 for discriminating patients with comorbid severe depressive symptoms. AUC, area under the curve.

## 4. Discussion

This study systematically investigated the prevalence and clinical relevance of SDS comorbidities in patients with first-episode SZ who did not receive medication. The findings indicate that the prevalence of SDS in this population was 49.7% (159/320). Multivariate analysis revealed that severe psychotic symptoms, anxiety symptoms, and suicide attempts were independently associated with comorbid SDS. These three factors appear to interact in influencing the onset and progression of SDS in patients with SZ, underscoring their significance for early identification and targeted intervention.

### 4.1 Prevalence and Comparison With Previous Studies

The prevalence of comorbid SDS observed in this study (49.7%) aligns with previously reported estimates [20]. Xi et al. [21] reported a high prevalence of depressive symptoms among Chinese patients with SZ, noting the frequent co-occurrence of depressive and anxiety symptoms, a finding consistent with other observations. A multi-national meta-analysis indicated that the overall prevalence of comorbidities with depressive symptoms in schizophrenia was 32.6% (95% CI 27.9–37.6, based on 18 studies and 6140 schizophrenia patients) [22].

However, the reported prevalence rates vary across studies. For instance, Buckley et al. [23] reported a comorbid depression prevalence of approximately 40% in patients with SZ, which is slightly lower than our estimate, whereas Buckley et al. [23] observed a higher prevalence of 50%. The main reasons for these differences may be: First, there are differences in the study population, including medication status, illness duration, and severity of primary psychopathology, which may contribute to variability. This study specifically focused on antipsychotic-naïve, first-episode patients, whereas other investigations included chronic SZ samples [24]; symptom profiles differ across illness stages, potentially affecting the rates of comorbidity with depression. Second, the heterogeneity in the assessment instruments and diagnostic criteria for SDS may influence the prevalence estimates. Several investigations employed the 17-item Hamilton Depression Rating Scale (HAMD-17) [25], whereas this study employed stricter classification criteria based on the 24-item version (HAMD-24), potentially affecting the case ascertainment. Third, regional and ethnic factors, including cultural background and environmental context, may influence mental health presentations. Notably, both Chen and Li [24] and the current study examined Chinese populations, yielding consistent prevalence estimates, thereby minimizing the heterogeneity attributable to cross-cultural differences.

### 4.2 Association With Psychotic Symptoms

In this relatively homogeneous sample, it was found that severe psychotic symptoms were independently associated with comorbid SDS, consistent with previous research [26]. Zhou et al. [27] demonstrated significant positive correlations between total PANSS scores, positive symptom subscale scores, and depression severity, indicating that greater psychotic symptom burden increases the risk of depression. Positive symptoms, particularly delusions and hallucinations, may induce emotional distress and demoralization, thereby elevating the risk of depression [2]. Furthermore, patients with severe psychotic symptoms often experience greater functional impairment and social withdrawal, which may exacerbate depressive symptoms [28]. Häfner et al. [29] similarly identified depression as a prominent clinical feature in early course SZ, closely linked to psychotic symptom severity.

The underlying neurobiological mechanism may involve dysfunction of the dopaminergic (DA) system. Patients with SZ typically exhibit DA hyperactivity, particularly in the mesolimbic system, which is implicated in the generation of positive symptoms, such as hallucinations and delusions [30]. Most antipsychotic medications alleviate these symptoms through dopamine D2 receptor antagonism [31]. However, DA dysfunction is also a core pathophysiological substrate of depression. The DA system critically regulates reward anticipation and hedonic experience [32], and anhedonia, a cardinal symptom of depression, is typically associated with DA hypofunction [33]. During SZ progression, hyperactive DA signaling in specific brain regions (e.g., mesolimbic system) generating psychotic symptoms may concurrently produce relative DA deficiency in other circuits (e.g., prefrontal cortex-striatal pathways) [34]. This “dysfunctional equilibrium” (imbalanced dopaminergic tone) may explain why some patients with SZ experience or worsening depressive symptoms despite remission of psychotic symptoms [2]. Consequently, although antipsychotics reduce hallucinations and delusions via D2 receptor blockade, this antagonism may further suppress DA signaling in prefrontal-striatal circuits, potentially inducing anhedonia and exacerbating depression [31].

### 4.3 Association With Anxiety Symptoms

Anxiety symptoms emerged as another independent predictor of comorbid SDS in SZ patients, consistent with a well-established anxiety-depression comorbidity extensively documented in the literature [35,36]. The incidence of anxiety symptoms is substantially elevated in SZ patients with depression, with anxiety often preceding or intensifying depressive episodes [37]. After controlling for confounding variables including illness duration, psychotic symptom severity, antipsychotic exposure, and social support, anxiety remained a robust predictor of comorbid depression, supporting its role as an independent etiological factor [38]. Large-scale Chinese clinical cohorts have demonstrated that anxiety symptoms in SZ patients correlate positively with depression risk, with higher anxiety levels predicting greater depression likelihood [36]. Clinically, anxiety symptoms amplify subjective distress and sleep disturbances and promote anhedonia, hopelessness, and depressive cognitions [39]. Consistent with a dimensional perspective on depressive symptomatology as a transdiagnostic feature across psychotic and affective disorders [40], our findings suggest that the high HAMA scores observed in the SDS group reflect a shared negative affectivity dimension rather than separable anxiety and depression constructs. Beyond the clinical predictors discussed above, neurobiological mechanisms involving the hypothalamic-pituitary-adrenal (HPA) axis may further explain the high comorbidity between anxiety and depressive symptoms in SZ patients. A substantial body of literature has documented HPA axis hyperactivity and cortisol dysregulation across psychotic and affective disorders [41,42]. Of particular relevance to the present findings, Monteleone and colleagues [41] demonstrated that chronic SZ patients whose illness onset occurred after cannabis exposure exhibited significantly elevated baseline morning salivary cortisol levels and a flattened cortisol awakening response (CAR) compared to healthy controls, whereas SZ patients without cannabis exposure showed no such abnormalities. Preclinical and clinical evidence indicates that chronic cortisol elevation can enhance mesolimbic dopamine release and sensitize dopamine receptors, while dopamine dysregulation in turn stimulates HPA activity, creating a mutual amplification loop between the two systems [43]. In our study, SZ patients with comorbid SDS (the “SDS group”) had a mean HAMA score of 34.84, indicating severe anxiety that likely reflects heightened negative affectivity and chronic stress load. Given the absence of direct biological markers (e.g., salivary or plasma cortisol, dopamine metabolites) in the current study, the proposed dopaminergic-HPA interactive framework should be interpreted as a heuristic conceptual model rather than a formally tested theory. Future longitudinal studies incorporating repeated measurements of cortisol, catecholamines, and inflammatory markers are warranted to empirically test this hypothesized pathway and to determine whether HPA dysregulation precedes or follows the onset of depressive comorbidity in SZ.

In the present study, patients with severe SDS exhibited more severe psychotic symptoms and higher anxiety scores than those with non-severe depression. This pattern may reflect a “mutual amplification effect” arising from DA system imbalance and HPA axis hyperactivity, which jointly promote SDS onset and progression. Mesolimbic DA pathway hyperactivity in patients with SZ intensifies psychotic symptoms, subsequently activating the HPA axis via neural projections between the prefrontal cortex and hypothalamus, resulting in excessive cortisol secretion. Chronic hypercortisolemia may further suppress prefrontal DA release, compromising the capacity of the prefrontal cortex to regulate emotion and cognition, thereby inducing or aggravating depressive symptoms.

### 4.4 Association With Suicide Attempt

Importantly, this study identified suicide attempts as being independently associated with SDS comorbidity in patients with SZ, highlighting the critical clinical significance of monitoring suicidal tendencies in this population. Taktak et al. [44] reported that patients with SZ with elevated anxiety and depression scores exhibited significantly greater suicide risk than those with lower scores, consistent with the findings reported here. In a study of 516 first-episode patients, a significant independent association was identified between the co-occurrence of anxiety and depression symptoms (i.e., negative affectivity) and suicide attempts. It was proposed that this particular symptom cluster represents not merely low mood but may substantially increase the psychological burden, potentially precipitating more severe suicidal behaviors [45].

The underlying mechanism may involve SDS-induced loss of interest in life, diminished self-evaluation and hopelessness, thereby triggering suicidal ideation and behavior [46,47]. Concurrent severe psychotic and anxiety symptoms may increase the psychological burden and severity of suicide attempts [3]. Patients with SZ, recognizing their functional impairment and poor prognosis, often experience elevated SDS, subsequently increasing the risk of suicide attempts, particularly prominent in early illness stages, as antipsychotic-naïve patients discharged after first-episode SZ demonstrate a 37-fold greater suicide mortality risk compared to the general population [48]. Additionally, DA system dysfunction and stress system activation may synergistically promote suicide attempts among these patients [49].

Several methodological limitations must be considered. First, this cross-sectional design cannot exclude the possibility that some patients experienced undiagnosed or subclinical depressive symptoms before suicide attempts. These potentially prodromal depressive symptoms may have served as underlying drivers of suicide attempts, subsequently progressing to full SDS. This uncertainty represents an inherent limitation of cross-sectional designs, which require verification through prospective longitudinal studies. Second, reverse causality (i.e., SDS leading to suicide attempts) cannot be excluded from this study. As cross-sectional measurements, the current SDS status reflects symptom severity at enrollment. The extant literature confirms SDS as one of the strongest predictors of suicidal behavior. Such findings suggest that the clinical assessment of patients with SZ should incorporate a systematic evaluation of depressive symptoms alongside close monitoring of suicidal tendencies, enabling timely targeted interventions to reduce suicide risk.

### 4.5 Other Variables and Study Limitations

Notably, this study did not identify significant associations between comorbid depressive symptoms and demographic variables, including sex, age, illness duration, or metabolic parameters, whereas prior studies have reported inconsistent findings regarding these associations [50,51,52]. These discrepancies may reflect sample characteristics, study design variations, or differences between antipsychotic-naïve first-episode patients and chronically medicated cohorts. The strengths of the present study include its relatively large sample size and the application of standardized tools for evaluating depression, anxiety, and psychotic symptoms.

The study also had further limitations. First, its cross-sectional nature prevented the establishment of causal relationships. This study adopted a cross-sectional design and was unable to infer the causal relationship between the four independent influencing factors and SDS. Therefore, the results of this study should be regarded as generating hypotheses rather than verifying causality. It is suggested that prospective longitudinal studies be conducted in the future to clarify the observed causal mechanisms associated with it through repeated measurements, thereby providing higher-level evidence for clinical practice. Second, this study failed to obtain data on other relevant confounding factors (such as substance use, cognitive function and social support). Therefore, we cannot control the confounding bias that these factors may cause, which may affect the robustness of the results. Subsequent studies will collect the set and adjust these variables to further verify the conclusions of this study. Third, the single-center study design may be subject to selection bias; as the sample may not fully represent regional or national populations of patients with SZ. Therefore, multicenter investigations with larger and more heterogeneous samples are warranted to validate these findings. Fourth, biological markers, such as inflammatory cytokines and neuroendocrine indicators (e.g., cortisol), were not assessed. Consequently, the mechanistic relationships between the three identified factors and comorbid SDS could not be explored in depth.

## 5. Conclusions

The findings reported here demonstrate that comorbid SDS is highly prevalent (49.7%) among patients with SZ. Severe psychotic symptoms, anxiety symptoms, and suicidal tendencies were independent predictors of SDS comorbidity in this population. These results have important clinical implications: Assessment and screening for SDS should be integrated into routine clinical care for patients with SZ, particularly among those with severe psychotic symptoms, prominent anxiety, or suicidal tendencies. Clinicians provide universal psychological education to patients and their families on the identification of depressive symptoms; routine evaluations were conducted every 3 months at baseline and within the first year of treatment using simple and validated tools (such as the Calgary Schizophrenia Depression Scale or Patient Health Questionnaire-9 (PHQ-9)); for those with clinically significant depressive or suicidal tendencies, targeted intervention should be carried out in a timely manner (such as cognitive behavioral therapy or low-dose antidepressants). Timely targeted interventions is expected to improve treatment outcomes and prognosis, while reducing adverse consequences, including suicide. We recommend routine depression screening in first-episode SZ, given that depressive comorbidity is common yet often overlooked due to overlapping negative or positive symptoms. Screening should be particularly rigorous in patients with severe psychotic symptoms, prominent anxiety, or suicidal tendencies. Future research should focus on elucidating the causal relationships and underlying mechanisms linking these predictors to comorbid SDS, thereby providing a stronger evidence base for personalized prevention and treatment strategies.

## Data Availability

The data used in this study are available from the corresponding authors upon reasonable requests.

## References

[1] Solmi M, Seitidis G, Mavridis D, Correll CU, Dragioti E, Guimond S (2023). Incidence, prevalence, and global burden of schizophrenia - data, with critical appraisal, from the Global Burden of Disease (GBD) 2019. Molecular Psychiatry.

[2] Conley RR, Ascher-Svanum H, Zhu B, Faries DE, Kinon BJ (2007). The burden of depressive symptoms in the long-term treatment of patients with schizophrenia. Schizophrenia Research.

[3] Bornheimer LA, Jaccard J (2017). Symptoms of Depression, Positive Symptoms of Psychosis, and Suicidal Ideation Among Adults Diagnosed With Schizophrenia Within the Clinical Antipsychotic Trials of Intervention Effectiveness. Archives of Suicide Research: Official Journal of the International Academy for Suicide Research.

[4] Krynicki CR, Upthegrove R, Deakin JFW, Barnes TRE (2018). The relationship between negative symptoms and depression in schizophrenia: a systematic review. Acta Psychiatrica Scandinavica.

[5] Shao X, Ren H, Li J, He J, Dai L, Dong M (2024). Intra-individual structural covariance network in schizophrenia patients with persistent auditory hallucinations. Schizophrenia (Heidelberg, Germany).

[6] Maini K, Gould H, Hicks J, Iqbal F, Patterson J, Edinoff AN (2021). Aripiprazole Lauroxil, a Novel Injectable Long-Acting Antipsychotic Treatment for Adults with Schizophrenia: A Comprehensive Review. Neurology International.

[7] Upthegrove R, Marwaha S, Birchwood M (2017). Depression and Schizophrenia: Cause, Consequence, or Trans-diagnostic Issue?. Schizophrenia Bulletin.

[8] Berardelli I, Sarubbi S, Rogante E, Hawkins M, Cocco G, Erbuto D (2019). The Role of Demoralization and Hopelessness in Suicide Risk in Schizophrenia: A Review of the Literature. Medicina (Kaunas, Lithuania).

[9] Rajkumar RP (2015). Depressive Symptoms during an Acute Schizophrenic Episode: Frequency and Clinical Correlates. Depression Research and Treatment.

[10] Tariku M, Ali T, Misgana T, Hajure M, Asfaw H (2021). Depression among Patients with Schizophrenia in Ethiopian Mental Health Hospital: Association with Sociodemographic and Clinical Variables: A Cross-Sectional Study. Depression Research and Treatment.

[11] Köhler S, van Os J, de Graaf R, Vollebergh W, Verhey F, Krabbendam L (2007). Psychosis risk as a function of age at onset: a comparison between early- and late-onset psychosis in a general population sample. Social Psychiatry and Psychiatric Epidemiology.

[12] Matsunaga M, Li Y, He Y, Kishi T, Tanihara S, Iwata N (2023). Physical, psychiatric, and social comorbidities of individuals with schizophrenia living in the community in Japan. International Journal of Environmental Research and Public Health.

[13] Dantzer R, O'Connor JC, Freund GG, Johnson RW, Kelley KW (2008). From inflammation to sickness and depression: when the immune system subjugates the brain. Nature Reviews. Neuroscience.

[14] Conring F, Gangl N, Derome M, Wiest R, Federspiel A, Walther S (2023). Associations of resting-state perfusion and auditory verbal hallucinations with and without emotional content in schizophrenia. NeuroImage. Clinical.

[15] Peng RJ, Sun C, Fan Y, Li J, Gao J, Sun WX (2025). Oxidative stress and cognitive function in Chinese patients with major depressive disorder: the mediating role of depression severity. BMC Psychiatry.

[16] Hamilton M (1959). The assessment of anxiety states by rating. The British Journal of Medical Psychology.

[17] Kay SR, Fiszbein A, Opler LA (1987). The positive and negative syndrome scale (PANSS) for schizophrenia. Schizophrenia Bulletin.

[18] Posner K, Oquendo MA, Gould M, Stanley B, Davies M (2007). Columbia Classification Algorithm of Suicide Assessment (C-CASA): classification of suicidal events in the FDA's pediatric suicidal risk analysis of antidepressants. The American Journal of Psychiatry.

[19] Kent State University (2018). Statistical & qualitative data analysis software: About GPower. https://libguides.library.kent.edu/statconsulting/GPower.

[20] Liu R, Fang X, Yu L, Wang D, Wu Z, Guo C (2022). Gender Differences of Schizophrenia Patients With and Without Depressive Symptoms in Clinical Characteristics. Frontiers in Psychiatry.

[21] Xi SJ, Shen MX, Wang Y, Zhou W, Xiao SY, Tebes JK (2021). Depressive symptoms, anxiety symptoms, and their co-occurrence among people living with schizophrenia in China: Prevalence and correlates. Journal of Clinical Psychology.

[22] Etchecopar-Etchart D, Korchia T, Loundou A, Llorca PM, Auquier P, Lançon C (2021). Comorbid Major Depressive Disorder in Schizophrenia: A Systematic Review and Meta-Analysis. Schizophrenia Bulletin.

[23] Buckley PF, Miller BJ, Lehrer DS, Castle DJ (2009). Psychiatric Comorbidities and Schizophrenia. Schizophrenia Bulletin.

[24] Chen Y, Li W (2021). Prevalence, Influencing Factors, and Cognitive Characteristics of Depressive Symptoms in Elderly Patients with Schizophrenia. Neuropsychiatric Disease and Treatment.

[25] Schennach R, Obermeier M, Seemüller F, Jäger M, Schmauss M, Laux G (2012). Evaluating depressive symptoms in schizophrenia: a psychometric comparison of the Calgary Depression Scale for Schizophrenia and the Hamilton Depression Rating Scale. Psychopathology.

[26] Li W, Yang Y, An FR, Zhang L, Ungvari GS, Jackson T (2020). Prevalence of comorbid depression in schizophrenia: A meta-analysis of observational studies. Journal of Affective Disorders.

[27] Zhou Y, Li Z, Wang Y, Huang H, Chen W, Dong L (2020). Prevalence and clinical correlates of psychotic depression in first-episode and drug-naïve outpatients with major depressive disorder in a Chinese Han population. Journal of Affective Disorders.

[28] Kehinde F, Bharmal AV, Goodyer IM, Kelvin R, Dubicka B, Midgley N (2022). Cross-sectional and longitudinal associations between psychotic and depressive symptoms in depressed adolescents. European Child & Adolescent Psychiatry.

[29] Häfner H, Maurer K, Trendler G, An der Heiden W, Schmidt M (2005). The early course of schizophrenia and depression*. European Archives of Psychiatry and Clinical Neuroscience.

[30] Oliver D, Chesney E, Cullen AE, Davies C, Englund A, Gifford G (2024). Exploring causal mechanisms of psychosis risk. Neuroscience and Biobehavioral Reviews.

[31] Meyer JM (2024). How antipsychotics work in schizophrenia: a primer on mechanisms. CNS Spectrums.

[32] Hird EJ, Slanina-Davies A, Lewis G, Hamer M, Roiser JP (2024). From movement to motivation: a proposed framework to understand the antidepressant effect of exercise. Translational Psychiatry.

[33] Guyer AE (2024). Understanding the paradox: anhedonia and the unexpected divergence from reward seeking during adolescence - A commentary on Gupta et al. (2024). Journal of Child Psychology and Psychiatry, and Allied Disciplines.

[34] Gee A, Dazzan P, Grace AA, Modinos G (2025). Corticolimbic circuitry as a druggable target in schizophrenia spectrum disorders: a narrative review. Translational Psychiatry.

[35] Tao Y, Zhao R, Yang B, Han J, Li Y (2024). Dissecting the shared genetic landscape of anxiety, depression, and schizophrenia. Journal of Translational Medicine.

[36] Chen S, Sun X, Zhu Q, Zhao Y, Tang J, Song H (2023). Factors influencing the level of depression and anxiety of community-dwelling patients with schizophrenia in China during the COVID-19 pandemic. International Journal of Environmental Research and Public Health.

[37] Kiran C, Chaudhury S (2018). Correlates and management of comorbid anxiety disorders in schizophrenia. Industrial Psychiatry Journal.

[38] Tabrizi F, Rosén J, Grönvall H, William-Olsson VR, Arner E, Magnusson PK (2025). Heritability and polygenic load for comorbid anxiety and depression. Translational Psychiatry.

[39] Gallagher MR, Collins AC, Lekkas D, Nemesure MD, Griffin TZ, Price GD (2025). Anhedonia in flux: Understanding the associations of emotion regulation and anxiety with anhedonia dynamics in a sample with major depressive disorder. Journal of Affective Disorders.

[40] Fiorillo A, Demyttenaere K, Martiadis V, Martinotti G (2025). Editorial: Treatment resistant depression (TRD): epidemiology, clinic, burden and treatment. Frontiers in Psychiatry.

[41] Monteleone P, Di Filippo C, Fabrazzo M, Milano W, Martiadis V, Corrivetti G (2014). Flattened cortisol awakening response in chronic patients with schizophrenia onset after cannabis exposure. Psychiatry Research.

[42] Berger M, Kraeuter AK, Romanik D, Malouf P, Amminger GP, Sarnyai Z (2016). Cortisol awakening response in patients with psychosis: Systematic review and meta-analysis. Neuroscience and Biobehavioral Reviews.

[43] P S, Vellapandian C (2024). Hypothalamic-Pituitary-Adrenal (HPA) Axis: Unveiling the Potential Mechanisms Involved in Stress-Induced Alzheimer's Disease and Depression. Cureus.

[44] Taktak Ş, Öz HS (2023). The Relationship Between Depression, Anxiety and Stress Levels on Suicidal Behavior in Patients with Schizophrenia. Psychiatry Clin Psychopharmacol.

[45] Salvatore P, Baldessarini RJ, Khalsa HMK, Indic P, Maggini C, Tohen M (2013). Negative Affective Features in 516 Cases of First Psychotic Disorder Episodes: Relationship to Suicidal Risk. Journal of Depression & Anxiety.

[46] Darquennes G, Wacquier B, Loas G, Hein M (2023). Suicidal Ideations in Major Depressed Subjects: Role of the Temporal Dynamics of Anhedonia. Brain Sciences.

[47] Riera-Serra P, Navarra-Ventura G, Castro A, Gili M, Salazar-Cedillo A, Ricci-Cabello I (2024). Clinical predictors of suicidal ideation, suicide attempts and suicide death in depressive disorder: a systematic review and meta-analysis. European Archives of Psychiatry and Clinical Neuroscience.

[48] Sher L, Kahn RS (2019). Suicide in Schizophrenia: An Educational Overview. Medicina (Kaunas, Lithuania).

[49] Rodríguez-Muñoz M, Sánchez-Blázquez P, Callado LF, Meana JJ, Garzón-Niño J (2017). Schizophrenia and depression, two poles of endocannabinoid system deregulation. Translational Psychiatry.

[50] Pan A, Keum N, Okereke OI, Sun Q, Kivimaki M, Rubin RR (2012). Bidirectional Association Between Depression and Metabolic Syndrome: A systematic review and meta-analysis of epidemiological studies. Diabetes Care.

[51] Bakhtiari A, Hashemi M, Hosseini SR, Omidvar S, Bijani A, Khairkhah F (2018). The Relationship between Depression and Metabolic Syndrome in the Elderly Population: The Cohort Aging Study. Iranian Journal of Psychiatry.

[52] Gjerde PB, Simonsen CE, Lagerberg TV, Steen NE, Andreassen OA, Steen VM (2021). Sex-Specific Effect of Serum Lipids and Body Mass Index on Psychotic Symptoms, a Cross-Sectional Study of First-Episode Psychosis Patients. Frontiers in Psychiatry.

